# Impact of COVID-19 on ischemic stroke patterns and outcomes: a multicenter retrospective study using propensity score matching

**DOI:** 10.3389/fmed.2026.1750243

**Published:** 2026-02-03

**Authors:** Daniyah A. Almarghalani, Khulood A. Almehmadi, Alaa M. Hammad, Mohammad S. Alzahrani, Marwa Qadri, Joud Amin Sindi, Rahaf Abdulaziz Alharthi, Maha Nasser Aloudah, Sarah Abdulrahman Alghamdi, Shahad Jameel Alsuwat, Muath B. Almutairi, Alqassem Y. Hakami, Faisal F. Alamri, Seraj Makkawi

**Affiliations:** 1 Department of Pharmacology and Toxicology, College of Pharmacy, Taif University, Taif, Saudi Arabia; 2 Stroke Research Unit, Taif University, Taif, Saudi Arabia; 3 Department of Pharmacology and Toxicology, Faculty of Pharmacy, King Abdulaziz University, Jeddah, Saudi Arabia; 4 Department of Pharmacy, Faculty of Pharmacy, Al-Zaytoonah University of Jordan, Amman, Jordan; 5 Department of Clinical Pharmacy, College of Pharmacy, Taif University, Taif, Saudi Arabia; 6 Department of Pharmacology and Toxicology, College of Pharmacy, Jazan University, Jazan, Saudi Arabia; 7 Health Research Center, Jazan University, Jazan, Saudi Arabia; 8 National Guard Health Affairs, King Salman Specialized Hospital, Taif, Saudi Arabia; 9 College of Medicine, King Saud Bin Abdulaziz University for Health Sciences, Jeddah, Saudi Arabia; 10 King Abdullah International Medical Research Center, Jeddah, Saudi Arabia; 11 Department of Basic Sciences, College of Science and Health Professions, King Saud bin Abdulaziz University for Health Sciences, Jeddah, Saudi Arabia; 12 Department of Neurology, Ministry of the National Guard-Health Affairs, Jeddah, Saudi Arabia

**Keywords:** COVID-19, ischemic stroke, mortality, propensity score matching, stroke outcomes

## Abstract

**Background:**

The impact of coronavirus disease 2019 (COVID-19) on ischemic stroke outcomes remains uncertain, particularly in multicenter Middle Eastern cohorts. This study aimed to assess stroke-related complications and in-hospital outcomes in patients with and without COVID-19 using a propensity score–matched design.

**Methods:**

We retrospectively analyzed 820 ischemic stroke patients admitted to three tertiary hospitals in Saudi Arabia between March 2020 and March 2021. Among these patients, 711 had no COVID-19, and 109 had confirmed COVID-19. Propensity score matching (2:1) was performed on the basis of age, sex, smoking status, diabetes status, hypertension status, and ischemic heart disease, resulting in a matched cohort of 327 patients (218 non-COVID-19 patients and 109 COVID-19 patients). Clinical outcomes were compared via conditional logistic regression.

**Results:**

After matching, COVID-19 patients had significantly longer hospital stays (median 5 vs. 3 days, *p* = 0.044) and higher rates of pneumonia (54.1% vs. 10.6%, *p* < 0.001), cognitive impairment (11.9% vs. 2.8%, *p* = 0.001), and in-hospital mortality (23.9% vs. 10.1%, *p* = 0.001). COVID-19 infection was significantly associated with pneumonia (OR = 10.88; 95% CI: 5.36–22.08, *p* < 0.001), cognitive impairment (OR = 5.81; 95% CI: 1.87–18.00, *p* = 0.002), and in-hospital death (OR = 2.98; 95% CI: 1.53–5.79, *p* = 0.001).

**Conclusion:**

COVID-19 infection independently worsens ischemic stroke outcomes, increasing the risk of pneumonia, cognitive impairment, and in-hospital mortality even after adjustment for baseline factors. These findings highlight the need for intensified respiratory and neurological monitoring and may guide the clinical prioritization of high-risk stroke patients during infectious disease outbreaks.

## Graphical abstract

**Figure:**
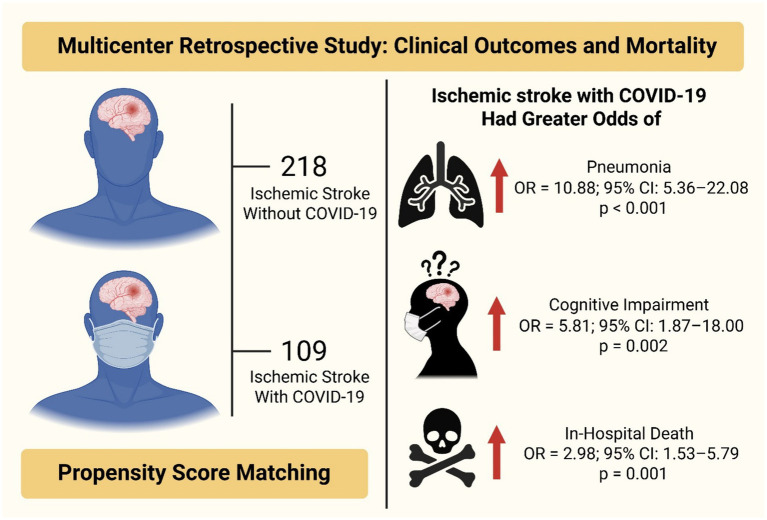
This was a multicenter retrospective study examining the clinical outcomes and mortality of ischemic stroke patients with and without COVID-19 after propensity score matching (*N* = 327: 218 without COVID-19, 109 with COVID-19). Patients with ischemic stroke and COVID-19 had greater odds of pneumonia (OR = 10.88, 95% CI: 5.36–22.08, *p* < 0.001), cognitive impairment (OR = 5.81, 95% CI: 1.87–18.00, *p* = 0.002), and in-hospital death (OR = 2.98, 95% CI: 1.53–5.79, *p* = 0.001) than those without COVID-19.

## 1 Introduction

Coronavirus disease 2019 (COVID-19), caused by severe acute respiratory syndrome coronavirus 2 (SARS-CoV-2), is associated with a range of multisystem complications, including acute cerebrovascular diseases. Although COVID-19 primarily affects the respiratory system, previous studies have established its ability to cause endothelial dysfunction, hypercoagulability, and systemic inflammation, all of which are key contributors to thromboembolic events, such as acute ischemic stroke (1). Several large cohort studies have shown that patients with both COVID-19 and ischemic stroke have significantly worse clinical outcomes than do stroke patients without COVID-19. For example, data from the U. S. National Inpatient Sample demonstrated higher in-hospital mortality in acute ischemic stroke patients with COVID-19 (16.9%) than in those without COVID-19 (4.1%) (2). This group exhibited a marked increase in mechanical ventilation usage, with elevated rates of acute venous thromboembolism, acute myocardial infarction, septic shock, cardiac arrest, and acute kidney injury and increased hospital stay durations and average total hospitalization costs (2). Similarly, Yaghi et al. reported that stroke patients with COVID-19 had greater stroke severity and D-dimer levels, increased rates of cryptogenic stroke and mortality, and worse functional outcomes than non-COVID-19 stroke patients did (3). A recent meta-analysis involving 43 studies and over 165,000 patients confirmed these findings, showing that patients with both acute ischemic stroke and COVID-19 had nearly fourfold higher mortality risk and worse functional outcomes than non-COVID-19 acute ischemic stroke patients did (4).

Emerging evidence also suggests that a prior history of stroke increases the risk of death in COVID-19-infected individuals, indicating a bidirectional relationship between cerebrovascular disease and SARS-CoV-2 infection (5). Several reports from the Middle East, such as a young Saudi who developed ischemic stroke following COVID-19 infection without any cerebrovascular risk factors (6), suggest unique regional characteristics that warrant further investigation. Previously, we reported that common comorbidities, such as hypertension, diabetes, and dyslipidemia, were more common in ischemic stroke patients with COVID-19 aged ≥65 years than in those aged <65 years and that pneumonia and dementia significantly predict mortality (7). However, these studies were limited by either a single-center design or cohorts that focused solely on age group differences in ischemic stroke patients with COVID-19, which may have introduced confounding factors due to baseline differences in age, sex, and comorbidities.

To address these limitations, advanced statistical methods, such as propensity score matching (PSM), are essential for balancing baseline characteristics and minimizing bias. PSM mimics the effects of randomization by matching participants on key covariates, allowing for a more accurate estimation of disease impact (8). This methodology has been effectively employed in stroke COVID-19 studies to provide more accurate comparisons between groups. For example, Harrison et al. used a large, matched cohort of ischemic stroke patients with and without COVID-19 and reported a significantly lower survival rate in the COVID-19 group and an adjusted 60-day mortality odds ratio of 2.51 (95% CI: 1.88–3.34) after PSM (9). Similarly, a global multicenter observational study by Ntaios et al. confirmed that ischemic stroke patients with COVID-19 had higher mortality and worse disability outcomes than did matched controls without COVID-19, even after adjusting for demographics and vascular risk factors via PSM (10). However, similar analyses remain scarce in the Saudi and Middle Eastern contexts, where demographic and health system factors may influence outcomes.

Therefore, this study aims to use a PSM design to compare the clinical outcomes and mortality rates between ischemic stroke patients with and without COVID-19 comprehensively. We hypothesized that, after adjusting for baseline comorbidities using propensity score matching, COVID-19 infection would remain independently associated with increased in-hospital mortality and complications in ischemic stroke patients.

## 2 Methods

### 2.1 Study design and setting

**Figure 1:**
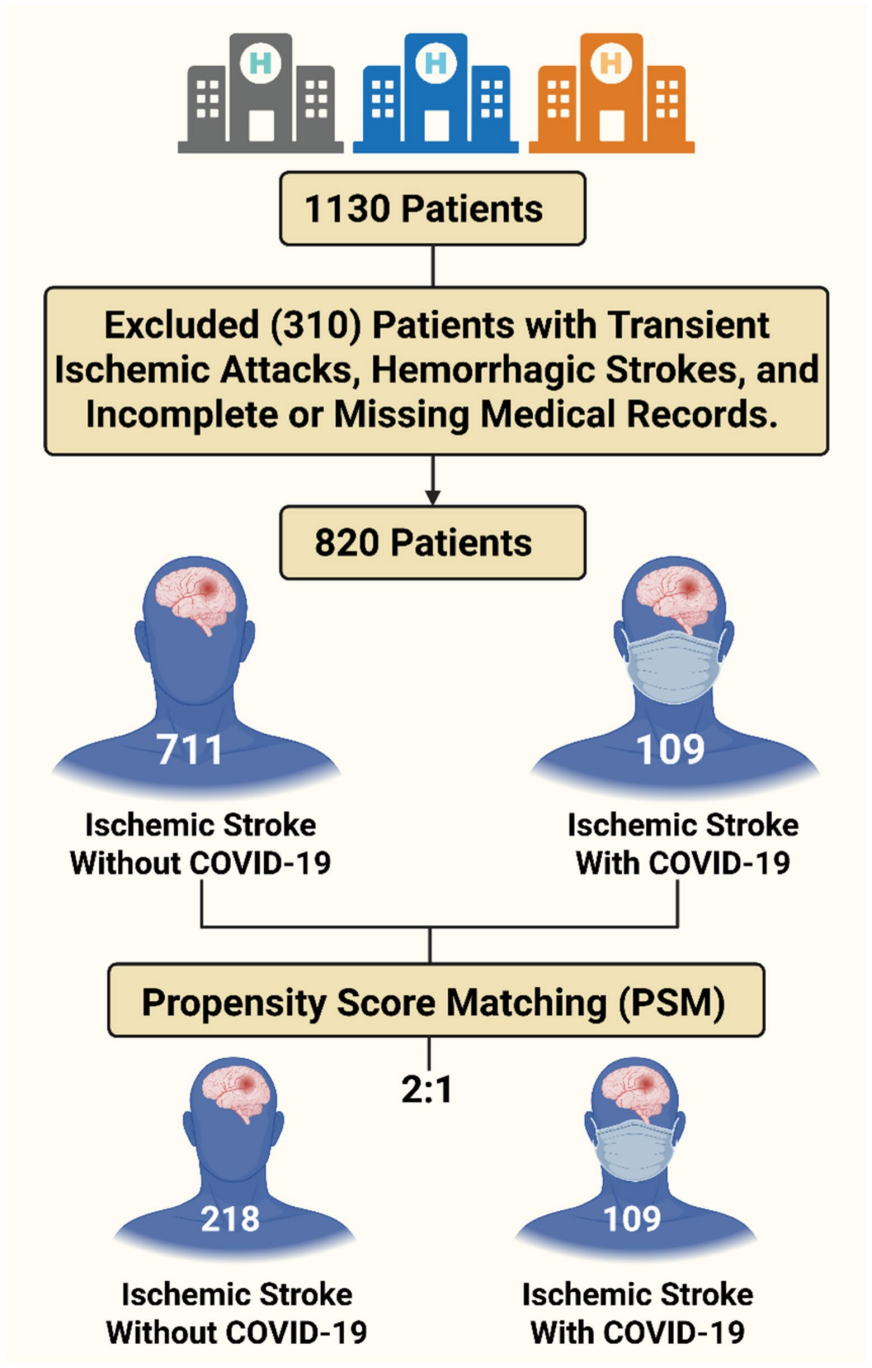
Flowchart illustrating the patient selection and matching process. A total of 1,130 ischemic stroke patients were initially identified from three tertiary care hospitals (multicenter cohort). After applying the predefined exclusion criteria, 820 patients were included in the study cohort, including 711 patients with ischemic stroke without COVID-19 and 109 with ischemic stroke and confirmed COVID-19 infection. Using propensity score matching at a 2:1 ratio (non-COVID-19: COVID-19), a final matched cohort of 327 patients was obtained, comprising 218 patients with ischemic stroke without COVID-19 and 109 with COVID-19. This matched cohort was used for comparative analysis of clinical characteristics, complications, and outcomes.

This retrospective observational cohort study was conducted at three tertiary care hospitals in Saudi Arabia [King Abdulaziz Medical City (KAMC) in Riyadh and Jeddah and Al-Hada Military Hospital in Taif] and compared stroke patients with and without confirmed COVID-19 infection. The study included patients admitted with a primary diagnosis of acute ischemic stroke between March 2020 and March 2021 (Figure 1 shows the flowchart of the study design). COVID-19 status was determined via polymerase chain reaction (PCR) at the time of hospital admission. The study received approval from the Institutional Review Board (IRB) of King Abdullah International Medical Research Center (KAIMRC) (IRB number 0603/23, dated March 5, 2023) and the Directorate of Health Affairs-Taif Research Ethics Committee (IRB number 773, dated December 23, 2022), adhering to the principles outlined in the Declaration of Helsinki.

### 2.2 Study population

Adult patients (aged ≥18 years) with acute ischemic stroke confirmed by neuroimaging techniques (e.g., magnetic resonance imaging (MRI) and/or computed tomography (CT) scans) were included in the analysis. The COVID-19 group comprised patients with laboratory-confirmed SARS-CoV-2 infection, whereas the non-COVID-19 group included stroke patients who tested negative for COVID-19 during the same period. Patients with hemorrhagic stroke (e.g., intracerebral hemorrhage or subarachnoid hemorrhage), transient ischemic attack (TIA), and incomplete data or missing outcome variables were excluded from the final analysis.

### 2.3 Data collection

Demographic data, clinical presentations, laboratory parameters, comorbidities, treatments, and outcomes were extracted from the hospital’s electronic medical records (BESTCare^®^ 2.0). The key variables included age, sex, body mass index (BMI), smoking status, comorbid conditions (diabetes mellitus, hypertension, ischemic heart disease (IHD), atrial fibrillation, and cancer), stroke severity as measured by the National Institutes of Health Stroke Scale (NIHSS), oxygen saturation, coagulation parameters [prothrombin time (PT) and partial thromboplastin time (PTT)], and length of hospital stay (LOS). Data on treatment modalities, including aspirin, clopidogrel, statins, tissue plasminogen activator (tPA), direct oral anticoagulants (DOACs), enoxaparin, and mechanical thrombectomy, were also collected. Anticoagulant use, including DOACs and enoxaparin, was recorded based on treatments administered or continued during the hospital stay for acute stroke management, as documented in medical records. Pre-hospitalization anticoagulant use was not available and thus not included in the analysis.

### 2.4 Outcome measures

Primary outcomes included comorbidities, management, length of hospital stay, in-hospital complications (pneumonia, cognitive impairment, deep vein thrombosis (DVT), hemorrhagic transformation, and stroke recurrence), and in-hospital death. These outcomes were compared between COVID-19 and non-COVID-19 stroke patients before and after propensity score matching.

### 2.5 Statistical analysis

Baseline characteristics of patients with and without COVID-19 were compared using independent-samples t tests for normally distributed continuous variables, Wilcoxon rank-sum tests for nonnormally distributed continuous variables, and chi-square tests or Fisher’s exact tests for categorical variables. To control for baseline differences and reduce confounding factors, a propensity score matching (PSM) approach was applied prior to the outcome analysis. Propensity scores were estimated via logistic regression based on key covariates: age, sex, smoking status, and comorbidities, including diabetes mellitus, hypertension, and ischemic heart disease. Optimal matching was implemented with a caliper of 0.2 on the logit scale, and a 1:2 ratio was used to pair each COVID-19-positive case with two COVID-19-negative controls. Matching quality was assessed via standardized mean differences (SMDs), with thresholds of 0.1 or less considered indicative of acceptable balance.

Following matching, unadjusted conditional logistic regression was performed to examine the associations between COVID-19 status and stroke-related complications and outcomes. The matched design was accounted for by stratifying the data into matched sets. Results were reported as odds ratios (ORs) with 95% confidence intervals (CIs), and statistical significance was defined as a two-sided *p* < 0.05. All analyses were conducted using SAS software, version 9.4 (SAS Institute Inc., NC, United States).

## 3 Results

### 3.1 Baseline characteristics before and after propensity score matching

Before PSM (*N* = 820: 711 non-COVID-19, 109 COVID-19), COVID-19 patients were older (69 ± 13.6 years vs. 65.7 ± 13.8 years, *p* = 0.022), had significantly lower PT and PTT (*p* < 0.001 and *p* = 0.003, respectively), and presented an increased prevalence of shortness of breath (61.5% vs. 2.9%, *p* < 0.001). Smoking was less common in COVID-19 patients (0.9% vs. 9%, *p* = 0.003), while their length of stay was longer (median 5 days vs. 3 days, *p* = 0.028). Additionally, cancer and enoxaparin use were more common in COVID-19 patients (*p* = 0.007 and *p* = 0.015, respectively).

After PSM, the matched groups were balanced on key variables, including age, sex, smoking status, diabetes mellitus status, hypertension status, and ischemic heart disease status, as confirmed by standardized mean differences. Most of the clinical variables were not significantly different after matching, indicating good covariate balance. However, PT and PTT remained significantly shorter in COVID-19 patients (*p* = 0.011 and *p* = 0.004, respectively), and shortness of breath remained markedly greater in the COVID-19 group (61.5% vs. 2.3%, *p* < 0.001). LOS was again significantly longer in COVID-19-positive patients (median 5 vs. 3 days, *p* = 0.044). Notably, cancer was more prevalent in the COVID-19 group (4.6%) than in the non-COVID-19 group (*p* = 0.004), and the use of enoxaparin and DOACs (direct oral anticoagulants) was significantly more common among COVID-19 patients (*p* = 0.011 and *p* = 0.036, respectively) (Table 1).

**Table 1:** Baseline characteristics of stroke patients with and without COVID-19 before and after propensity score matching (PSM).

Characteristics	Before PSM			After PSM		
	COVID-19 status			COVID-19 status		
	Non-COVID-19 (*N* = 711)	COVID-19 (*N* = 109)	*p*	Non-COVID-19 (*N* = 218)	With COVID-19 (*N* = 109)	*p*
Age ^†^	65.7 ± 13.8	69 ± 13.6	0.022	68.5 ± 12.3	69 ± 13.6	0.744
Male sex ^†^	455 (64)	67 (61.5)	0.609	135 (61.9)	67 (61.5)	0.936
BMI	28.4 ± 5.2	28.6 ± 5.1	0.757	29.1 ± 5.2	28.6 ± 5.1	0.561
O₂ saturation	97.6 ± 2.1	97.9 ± 1.7	0.167	97.5 ± 1.8	97.9 ± 1.7	0.144
PT (sec)	15.5 ± 7.0	13.7 ± 3.5	<0.001	15.0 ± 5.2	13.7 ± 3.5	0.011
PTT (sec)	52.1 ± 25.9	43.6 ± 11.8	0.003	53.7 ± 27.2	43.6 ± 11.8	0.004
SOB	21 (2.9)	67 (61.5)	<0.001	5 (2.3)	67 (61.5)	<0.001
Smoking (yes) ^†^	64 (9.0)	1 (0.9)	0.003	2 (0.9)	1 (0.9)	0.999
Admission NIHSS score	4.9 ± 4.2	5.2 ± 4.5	0.375	4.7 ± 4.3	5.2 ± 4.5	0.153
LOS (days)	3 (2–6)	5 (2–10)	0.028	3 (2–6)	5 (2–10)	0.044
Comorbidities						
Hypertension ^†^	421 (59.2)	70 (64.2)	0.321	132 (60.6)	70 (64.2)	0.519
Diabetes mellitus ^†^	418 (58.8)	65 (59.6)	0.868	125 (57.3)	65 (59.6)	0.692
IHD ^†^	84 (11.8)	20 (18.3)	0.056	41 (18.8)	20 (18.4)	0.920
Atrial fibrillation	27 (3.8)	3 (2.6)	0.588	5 (2.3)	3 (2.6)	0.801
Cancer	8 (1.1)	5 (4.6)	0.007	0 (0)	5 (4.6)	0.004
Management						
Aspirin use	540 (75.9)	73 (66.9)	0.044	154 (70.6)	73 (66.9)	0.497
Clopidogrel use	328 (46.1)	51 (46.8)	0.898	97 (44.5)	51 (46.8)	0.695
Statin use	507 (71.3)	74 (67.9)	0.465	154 (70.6)	74 (67.9)	0.609
tPA use	18 (2.5)	3 (2.8)	0.892	7 (3.2)	3 (2.8)	0.823
DOAC use	24 (3.4)	8 (7.3)	0.047	5 (2.3)	8 (7.3)	0.036
Enoxaparin use	182 (25.6)	40 (36.7)	0.015	51 (23.4)	40 (36.7)	0.011
Mechanical thrombectomy	7 (0.9)	2 (1.8)	0.428	4 (1.8)	2 (1.8)	0.999

Continuous variables are presented as the means ± SDs or medians (IQRs), depending on the distribution and test used. Categorical variables are presented as n (%). ^†^Variables included in the propensity score matching. PSM, propensity score matching; BMI, body mass index; SD, standard deviation; IQR, interquartile range; PT, prothrombin time; PTT, partial thromboplastin time; SOB, shortness of breath; NIHSS, National Institutes of Health Stroke Scale; LOS, length of stay; IHD, ischemic heart disease; tPA, tissue plasminogen activator; DOAC, direct oral anticoagulant.

### 3.2 Clinical complications and in-hospital outcomes before and after propensity score matching

Before and after PSM, significant differences were observed in both clinical complications and outcomes. Before matching, COVID-19 patients had a significantly greater incidence of pneumonia (54.1% vs. 7.3%, *p* < 0.001), cognitive impairment (11.9% vs. 1.8%, *p* < 0.001), and deep vein thrombosis (DVT; 7.3% vs. 3.2%, *p* = 0.036). In-hospital mortality was also notably higher among COVID-19 patients (23.9%) than among non-COVID-19 patients (7.6%, *p* < 0.001). After matching, the difference in DVT was no longer statistically significant; however, pneumonia (54.1% vs. 10.6%, *p* < 0.001), cognitive impairment (11.9% vs. 2.8%, *p* = 0.001), and in-hospital death (23.9% vs. 10.1%, *p* = 0.001) remained significantly higher in the COVID-19 group (Table 2).

**Table 2:** Comparison of stroke-related complications and outcomes between patients with and without COVID-19.

Variables	Before PSM			After PSM		
	COVID-19 status			COVID-19 status		
	Non-COVID-19 (*N* = 711)	COVID-19 (*N* = 109)	*p*	Non-COVID-19 (*N* = 218)	COVID-19 (*N* = 109)	*p*
Pneumonia, n (%)	52 (7.3)	59 (54.1)	<0.001	23 (10.6)	59 (54.1)	< 0.001
Cognitive impairment, n (%)	13 (1.8)	13 (11.9)	<0.001	6 (2.8)	13 (11.9)	0.001
Deep vein thrombosis (DVT), n (%)	23 (3.2)	8 (7.3)	0.036	9 (4.1)	8 (7.3)	0.218
Hemorrhagic transformation, n (%)	23 (3.2)	3 (2.8)	0.788	7 (3.2)	3 (2.8)	0.821
Stroke recurrence, n (%)	62 (8.7)	7 (6.4)	0.421	27 (12.4)	7 (6.4)	0.096
In-hospital death, n (%)	54 (7.6)	26 (23.9)	<0.001	22 (10.1)	26 (23.9)	0.001

### 3.3 Logistic regression analysis of outcome associations

**Figure 2:**
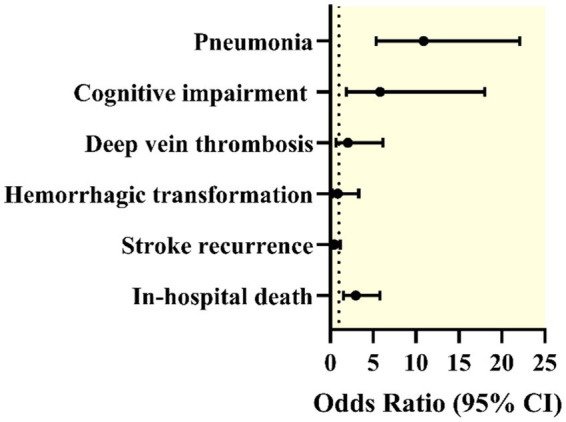
Forest plot displaying odds ratios (ORs) with 95% confidence intervals (CIs) for various complications in patients with both ischemic stroke and COVID-19 compared with those without COVID-19 after propensity score matching. The outcomes included pneumonia, cognitive impairment, deep vein thrombosis, hemorrhagic transformation, stroke recurrence, and in-hospital death.

Unadjusted conditional logistic regression analysis further supported these findings. COVID-19 infection was significantly associated with an increased risk of pneumonia (odds ratio (OR) = 10.88; 95% confidence interval (CI): 5.36–22.08; *p* < 0.001) and cognitive impairment (OR = 5.81; 95% CI: 1.87–18.0; *p* = 0.002). COVID-19 was also significantly associated with an increased risk of in-hospital mortality (OR = 2.98; 95% CI: 1.53–5.79; *p* = 0.001). No statistically significant associations were found for DVT (OR = 2.06; *p* = 0.194), stroke recurrence (OR = 0.50; *p* = 0.111), or hemorrhagic transformation (OR = 0.86; *p* = 0.823) (Table 3, Figure 2).

**Table 3:** Association between COVID-19 status and clinical outcomes (unadjusted conditional logistic regression).

Outcome	OR	95% CI	*p*
Pneumonia	10.88	5.36–22.08	<0.001
Cognitive impairment	5.81	1.87–18	0.002
Deep vein thrombosis (DVT)	2.06	0.69–6.15	0.194
Hemorrhagic transformation	0.86	0.22–3.32	0.823
Stroke recurrence	0.5	0.21–1.17	0.111
In-hospital death	2.98	1.53–5.79	0.001

OR, odds ratio; CI, confidence interval.

## 4 Discussion

This multicenter propensity score–matched study provides the first evidence from Saudi Arabia that COVID-19 significantly worsens in-hospital outcomes among patients with acute ischemic stroke. After controlling for major comorbidities, stroke patients with COVID-19 experienced longer hospitalizations and markedly higher rates of pneumonia, cognitive impairment, and mortality compared to those without COVID-19. These associations remained significant after matching, indicating that COVID-19 has an independent effect on stroke outcomes. Our findings are consistent with earlier reports showing that stroke patients who contract SARS-CoV-2 tend to experience higher rates of complications and mortality (11–14).

In the present analysis, pneumonia occurred far more frequently among stroke patients with COVID-19 than among those without it (54.1% versus 10.6%, respectively) (*p* < 0.001). This striking difference illustrates the combined impact of respiratory compromise and cerebrovascular injury. The inflammatory and endothelial disturbances triggered by COVID-19, along with weakened immune defenses, likely contribute to the increased risk of respiratory infections in this group (15–17). The strong association between pneumonia and COVID-19 observed in our conditional logistic regression model (OR = 10.88) underscores the need for vigilant monitoring of pulmonary function and for early preventive measures in patients at risk.

Notably, cognitive impairment was also more common among stroke patients with COVID-19, at 11.9%, than among non-COVID-19 patients, at 2.8% (*p* = 0.001). This pattern suggests that SARS-CoV-2 infection may aggravate poststroke neurological dysfunction. Mechanistically, systemic inflammation, coagulation abnormalities, and potential direct neurotropic effects of the virus may intensify ischemic injury and interfere with neuronal recovery (18–20). These observations align with prior studies that reported an increased occurrence of delirium, encephalopathy, and long-term cognitive decline among stroke patients infected with COVID-19 (21, 22).

In-hospital mortality was also substantially higher in the COVID-19 group, reaching 23.9% compared with 10.1% among noninfected stroke patients (*p* = 0.001). This finding mirrors international data showing a two- to fourfold increase in mortality rates among stroke patients with concurrent COVID-19 (21, 22). The odds ratio of 2.98 for in-hospital death reflects the profound effect of COVID-19 on clinical outcomes and highlights the urgency of tailoring management approaches to reduce fatality rates. Importantly, these differences persisted even after adjusting for baseline characteristics and comorbidities, implying that the infection itself plays a direct pathogenic role. Supporting studies have also linked the severity of COVID-19 and prolonged hospitalization with inflammatory and biochemical disturbances, such as elevated neutrophil counts and altered renal parameters, that may worsen neurological outcomes (23).

While no significant postmatching differences were detected in deep vein thrombosis, hemorrhagic transformation, or stroke recurrence, prematching comparisons suggested a higher rate of DVT in the COVID-19 group, which was consistent with the hypercoagulable state induced by the infection (24). The application of propensity score matching in our analysis was therefore essential to minimize confounding factors such as age, comorbidities, and baseline stroke severity and to provide a clearer understanding of how COVID-19 independently influences stroke prognosis.

Collectively, these findings have important clinical implications. Stroke patients who contract COVID-19 should be closely monitored for respiratory complications and cognitive decline, with early interventions aimed at reducing these risks. Clinicians should also consider enhanced supportive and multidisciplinary care strategies to improve survival and neurological recovery. Finally, our results reinforce the importance of preventive measures, including vaccination and infection control, among individuals at high risk for cerebrovascular disease, particularly older adults and those with multiple chronic conditions.

## 5 Limitations

This study has several key limitations. First, the retrospective design may introduce selection bias and residual confounding, even when propensity score matching is applied. Second, electronic medical records were obtained from three hospitals and may therefore exhibit variability in documentation practices, stroke management guidelines, and COVID-19 testing protocols. Third, the study accounted for only in-hospital outcomes and did not assess long-term functional recovery, disability, or postdischarge mortality. Additionally, COVID-19 severity and treatment data (e.g., use of antiviral agents, corticosteroids, or the need for ventilatory support) were not uniformly reported and therefore could not be incorporated into the analysis. The centers employed varying clinical indicators for COVID-19 severity (e.g., based on oxygen requirements or respiratory symptoms), but no uniform scale was used. While precise mild/moderate/severe classification is not possible retrospectively, the hospitalization requirement and high rates of complications (e.g., pneumonia in 54.1%) suggest the COVID-19 stroke group predominantly represented moderate-to-severe cases, which may limit direct comparisons with series including milder infections. Vaccination status was not addressed. The stroke subtype classification (using the TOAST criteria) was not available. We lacked imaging-based severity or perfusion data to explore differences in stroke mechanisms. While a larger cohort would enhance the robustness of our observations, the present study captured all consecutive eligible patients across three hospitals during the specified period, representing the totality of available cases in our setting at that time. The findings should thus be interpreted in the context of this real-world consecutive sample. Finally, cognitive impairment was determined by clinical reports rather than by standardized neuropsychological testing, which may limit its reliability. This assessment occurred in the acute stroke setting, where confounding factors such as pneumonia and other infections, medication side effects, altered sleep cycles, and delirium could contribute to transient cognitive changes, potentially reducing the accuracy and specificity of the diagnosis. Future research should investigate the long-term neurocognitive effects of COVID-19 in stroke patients, incorporate biomarker profiling, and examine whether vaccination or antiviral therapy affects outcomes.

## 6 Conclusion

This multicenter propensity score–matched study demonstrated that COVID-19 independently worsens in-hospital outcomes among patients with acute ischemic stroke in Saudi Arabia. Even after controlling for major confounders, COVID-19 was significantly associated with increased risks of pneumonia, cognitive impairment, prolonged hospitalization, and in-hospital mortality. These findings underscore the compounding effect of SARS-CoV-2 infection on cerebrovascular disease, highlighting the importance of early respiratory management, cognitive monitoring, and comprehensive multidisciplinary care in this high-risk population. Future multicenter, longitudinal studies integrating stroke subtype analysis, biomarker profiling, and vaccination data are warranted to elucidate the biological mechanisms underlying these poor outcomes and to guide the development of preventive and therapeutic strategies tailored for stroke patients in infectious disease settings.

## Data Availability

The raw data supporting the conclusions of this article will be made available by the authors, without undue reservation.

## Glossary

COVID-19: Coronavirus disease 2019 (COVID-19)
CI: Confidence interval
OR: Odds ratio
aOR: Adjusted odds ratio
PSM: Propensity score matching
KAMC: King Abdulaziz Medical City
PCR: Polymerase chain reaction
IRB: Institutional Review Board
KAIMRC: King Abdullah International Medical Research Center
BMI: Body mass index
MRI: Magnetic resonance imaging
CT: Computed tomography
TIA: Transient ischemic attack
IHD: Ischemic heart disease
NIHSS: National Institutes of Health Stroke Scale
PT: Prothrombin time
PTT: Partial thromboplastin time
tPA: Tissue plasminogen activator
LOS: Length of stay
DOAC: Direct oral anticoagulants
SMDs: Standardized mean differences
DVT: Deep vein thrombosis
SOB: Shortness of breath
IQRs: Interquartile ranges
SAS: Statistical analysis system
SD: Standard deviation
NC: North Carolina
USA: United States of America
TOAST: Trial of ORG 10172 in Acute Stroke Treatment
